# CRISPR-based gene knockout screens reveal deubiquitinases involved in HIV-1 latency in two Jurkat cell models

**DOI:** 10.1038/s41598-020-62375-3

**Published:** 2020-03-24

**Authors:** Anurag Rathore, Sho Iketani, Pengfei Wang, Manxue Jia, Vincent Sahi, David D. Ho

**Affiliations:** 1 Aaron Diamond AIDS Research Center, Columbia University Irving Medical Center, New York, NY 10032 USA; 2 Department of Microbiology and Immunology, Columbia University Irving Medical Center, New York, NY 10032 USA

**Keywords:** Target identification, Target identification, Functional genomics, Functional genomics

## Abstract

The major barrier to a HIV-1 cure is the persistence of latent genomes despite treatment with antiretrovirals. To investigate host factors which promote HIV-1 latency, we conducted a genome-wide functional knockout screen using CRISPR-Cas9 in a HIV-1 latency cell line model. This screen identified IWS1, POLE3, POLR1B, PSMD1, and TGM2 as potential regulators of HIV-1 latency, of which PSMD1 and TMG2 could be confirmed pharmacologically. Further investigation of PSMD1 revealed that an interacting enzyme, the deubiquitinase UCH37, was also involved in HIV-1 latency. We therefore conducted a comprehensive evaluation of the deubiquitinase family by gene knockout, identifying several deubiquitinases, UCH37, USP14, OTULIN, and USP5 as possible HIV-1 latency regulators. A specific inhibitor of USP14, IU1, reversed HIV-1 latency and displayed synergistic effects with other latency reversal agents. IU1 caused degradation of TDP-43, a negative regulator of HIV-1 transcription. Collectively, this study is the first comprehensive evaluation of deubiquitinases in HIV-1 latency and establishes that they may hold a critical role.

## Introduction

HIV-1 infection can be durably suppressed by combination antiretroviral therapy (cART), the current standard of treatment^1^. Yet, despite lowering viral load to undetectable levels, treatment is not curative, resulting in rapid rebound of the virus upon cessation of treatment^2^. Viral rebound occurs from a long-lived population of latently HIV-1 infected cells, primarily in resting CD4^+^ T cells^3^. These latent cells harbor transcriptionally silent proviral DNA that serve as a reservoir for the virus^4^. This reservoir must be purged for a cure to be achieved and therefore remains a major focus of current research efforts.

Transcriptional silencing of HIV-1 occurs through a multitude of mechanisms, of which several have been investigated in-depth^5^. Briefly, HIV-1 DNA can become latent as a consequence of its integration site^6^, epigenetic factors^7^, and/or as a result of cellular host factors^8^. Examples include the heterochromatinization of DNA, which inhibits accessibility of the HIV-1 promoter^9^, and sequestration of transcriptional factors such as NFκB that induce viral transcription^10^. Accordingly, significant effort has been spent on the identification of latency reversal agents (LRAs) that target such latency factors, with the aim of reactivating the virus for purging^11,12^. LRAs targeting many of these latency factors have been well-established, such as romidepsin, a histone deacetylase inhibitor (HDACi), which reverses heterochromatinization, and bryostatin, which targets the NFκB signaling pathway for reactivation^12^. Yet, although many LRAs have been successful *in vitro* in reactivating latent HIV-1^13^, those that have been taken to clinical trials have failed to show significant effects^14,15^. This may have been due to the suboptimal concentration of the LRAs *in vivo* or as yet unknown factors^16–18^. Such efforts have made it clear that HIV-1 latency involves a complex network of mechanisms that interplay with each other, and that additional pathways may need to be discovered in order to achieve successful reversal of latency.

Many investigations into host factors that play a role in HIV-1 latency have been conducted over the past several years, with the goal that additional insights could lead to the development of novel LRAs. The development of short hairpin RNA (shRNA), and, more recently, clustered regularly interspersed short palindromic repeats (CRISPR) and CRISPR-associated protein 9 (CRISPR-Cas9) methodologies, the latter of which has been utilized in several efforts to eradicate the HIV-1 latent reservoir by editing out the viral genome^19^ or by transplanting CRISPR-edited CCR5-null stem cells^20^, has allowed for systematic identification of such factors through loss-of-function screens^21–28^. These approaches benefit from the unbiased nature of such a screen, allowing for new pathways to be discovered. Examples include the work of Besnard *et al*., who uncovered mammalian target of rapamycin (mTOR) as a regulator of HIV-1 latency by using a pooled shRNA screen in the J-Lat 5A8 cell line (an HIV-1 latency model)^24^. Another group conducted a genome-wide shRNA screen in a different HIV-1 latency reporter cell line, 2D10, identifying accumulation of estrogen receptor-1 (ESR-1) on the HIV-1 promoter to be involved in HIV-1 latency^26^. Recent CRISPR-based approaches include a report by Li *et al*. that showed enrichment of single guide RNAs (sgRNAs) in 2D10 cells in a CRISPR interference (CRISPRi) screen that targeted suppressors of HIV-1 transcription (NFKBIA and CYLD), proteasome subunits (PSMD1, PSMD3, PSMD8) and a protein found in a transcriptional corepressor complex with HDAC1 (GON4L)^22^. In another example, Huang *et al*. used a CRISPR-Cas9 knockout screen targeting nuclear proteins in the J-Lat A2 cell line to identify MYC-induced nuclear antigen 53 (MINA53) as a novel latency promoting gene, possibly by altering methylation levels^21^. These approaches support the validity of genome-wide approaches of identifying HIV-1 latency factors, yet also underscore that significant differences are observed depending on the approach and HIV-1 latency model used, and that additional work must be done to fully understand the intricacies of HIV-1 latency.

Here, to identify novel classes of latency promoting factors, we conducted a CRISPR-Cas9-based whole genome-wide functional knockout screen. This initial screen was done in the J-Lat 10.6 cell line, a model for HIV-1 latency^29^. We found that sgRNAs targeting genes involved in RNA degradation and ubiquitin mediated proteolysis were enriched in reactivated cells, suggesting their involvement in maintenance of HIV-1 latency. Further gene enrichment and network analysis highlighted a potential network of HIV-1 latency factors, which were then verified in an additional HIV-1 latency cell line, resulting in the identification of the genes IWS1, POLE3, POLR1B, PSMD1, and TGM2 as potentially involved in HIV-1 latency. Furthermore, PSMD1, a subunit of the 19S proteasome and an adapter for the deubiquitinase complex showed significant HIV-1 latency reversal upon both genetic and pharmacologic inhibition. We then probed the role of UCH37 in HIV-1 latency, as it was known to be a deubiquitinase associated with the proteasome via PSMD1, finding that it too was a HIV-1 latency factor. This result fueled an investigation of the role of other deubiquitinases in HIV-1 latency. We systematically conducted an additional CRISPR-Cas9 knockout screen using individually designed sgRNAs against the deubiquitinating enzyme (DUB) family. This revealed previously known (CYLD, A20) as well as novel (USP5, USP14, and OTULIN) DUBs as HIV-1 latency promoting factors. Pharmacological modulation of USP14 with an inhibitor, IU1, was able to induce HIV-1 latency reversal without causing global T cell activation. Furthermore, IU1 exhibited a synergistic effect with currently available LRAs. This inhibitor may function through degradation of TAR DNA binding protein 43 (TDP43), a repressor of HIV-1 transcription. Collectively, we have identified several factors which may be involved in HIV-1 latency, and we propose that targeting such factors may serve as novel avenues for HIV-1 latency reversal.

## Results

### Genome-wide CRISPR-Cas9 knockout screen in J-Lat 10.6 cells

To systematically identify human genes that preserve HIV-1 latency, we performed a pooled genome-wide CRISPR-Cas9 knockout screen in J-Lat 10.6 cells, a widely used post-integration latency model that contains a single integrated replication-incompetent HIV-1 with a GFP reporter gene, such that GFP expression correlates with HIV-1 reactivation. This cell line was chosen for two reasons: (1) within currently available cell models, it has the highest reactivation response to latency reversal agents (LRAs) suggesting it may allow for weaker host factors to be identified, and (2) genome-wide screens had not been conducted with these cells.

**Figure 1:**
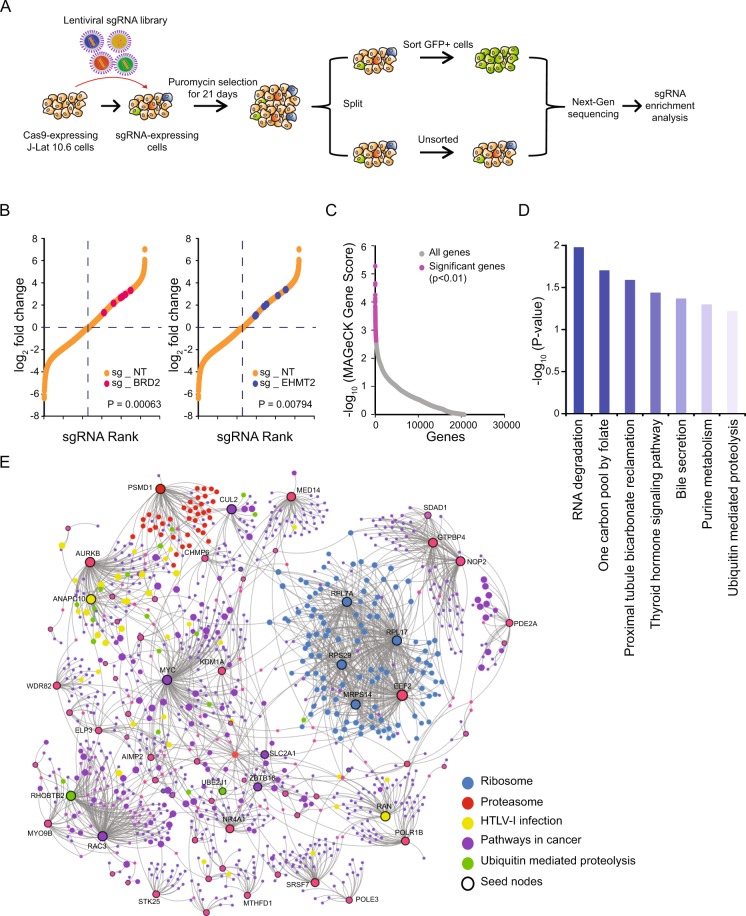
Genome-wide CRISPR-Cas9 KO screen in human cells identifies regulators of HIV-1 latency. (**A**) Schematic of the CRISPR-Cas9 screen. Cas9-expressing J-Lat 10.6 cells were transduced with lentiviruses expressing the sgRNA GeCKO V2 library (6 sgRNAs per gene). After 21 days of puromycin selection, the population was split in two, with half used for sorting GFP-positive (reactivated HIV-1) cells and the rest left unsorted. Both sorted and unsorted cells were then subjected to deep sequencing and analysis. The screen was repeated independently two times. (**B**) Enrichment of sgRNAs targeting latency-associated genes in sorted cells. Individual sgRNAs from the sorted GFP-positive cells were compared to sgRNAs from the unsorted population. Differences in enrichment were calculated and are represented as log_2_-normalized Fold Change (log_2_FC). Previously identified HIV-1 latency factors were examined to validate the overall approach; BRD2 and EHMT2 are shown as examples. Each of the six individual sgRNAs for the two genes are highlighted in red or blue, with the non-targeting control sgRNAs shown in orange. (**C**) Positively selected genes were identified by MAGeCK. Each gene was scored based on sgRNA frequencies across both replicates and are represented as −log_10_MAGeCK Gene Score in descending order. Genes with significant scores (n = 211, *P* < 0.01) across both replicates are highlighted in pink. (**D**) Identified hits were classified by using the Kyoto Encyclopedia of Genes and Genomes (KEGG) database (2019 version) to reveal overrepresented pathways. Each of the significantly enriched pathways (*P* < 0.05) are shown here as log-transformed *P* values. (**E**) Protein-protein interaction (PPI) network of the significantly enriched genes. These genes (n = 211) were analyzed in NetworkAnalyst to visualize gene interactions and to identify critical genes. A first order interaction network using the STRING interactome resulted in 1089 nodes, 1644 edges, and 70 seeds. Candidate genes for further analysis were then identified from this analysis based on two widely used topological measures, degree and betweenness centrality (see also Supplementary Data 4).

We first generated a J-Lat 10.6 variant which stably expressed functional *Streptococcus pyogenes* Cas9 (SpCas9) to conduct the genome-wide CRISPR-Cas9 knockout screen (referred to as J-Lat 10.6_Cas9). This cell line was then stably transduced with the GeCKO v2 sgRNA library, which contained 123,411 unique sgRNAs targeting 19,052 genes (6 sgRNAs per gene) along with 1000 non-targeting controls^30^. Cells were selected for with puromycin for 21 days before being split in half. Viable GFP-expressing cells were sorted from one half of the cells by flow cytometry, while the other half was left unsorted and served as a control (Fig. 1A). As the integrated HIV-1 in J-Lat 10.6 is transcriptionally silent at basal levels (<2% of cells are GFP+), we hypothesized that these enriched GFP-expressing cells would have knockouts of genes which maintained latency.

The sgRNAs found in both populations was quantified by isolating genomic DNA and then PCR amplifying and massively parallel sequencing the sgRNA-encoding cassettes. The frequency of each sgRNA was determined by MAGeCK (model-based analysis of genome wide CRISPR–Cas9 knockout) software^31^ (Supplementary Data 1). To confirm that the screen functioned as intended, we looked for the enrichment of sgRNA targeting host factors previously reported to be involved in HIV-1 latency. BRD2 and EHMT2, two genes which have previously been shown to be involved in HIV-1 transcriptional silencing had enrichment of all six sgRNAs in the sorted GFP-expressing population relative to the controls, indicating that the screen could be used for identification of HIV-1 latency factors^32,33^ (Fig. 1B).

To rank the performance of individual genes, both GeCKO A and B libraries were analyzed using MAGeCK to calculate the P-value and false discovery rate (FDR) for each gene. After filtering out non-protein-coding genes and predicted or under characterized genes, we identified 211 genes which were significantly enriched (P < 0.01), shown in pink in Fig. 1C. The rank and FDR of each gene in the latency screens are summarized in Supplementary Data 2.

We then determined the biological classification of these top 211 genes by using Enrichr for pathway enrichment analysis^34^. The genes were mapped into 139 KEGG reference pathways, of which, several were significantly enriched (P < 0.05). These pathways, which were finding enrichment in RNA degradation (n = 4), one carbon pool by folate (n = 2), proximal tubule bicarbonate reclamation (n = 2), thyroid hormone signaling pathway (n = 4), bile secretion (n = 3), purine metabolism (n = 4), and ubiquitin mediated proteolysis (n = 4), therefore may have a role in HIV-1 latency (Fig. 1D, Supplementary Data 3).

We next sought to determine if the observed pathways were related to one another. This was examined by using NetworkAnalyst, which creates networks based on a framework of known protein−protein interactions (PPI) captured in publicly curated databases^35^. First-order networks were constructed by incorporating the 211 genes from the screen as seed nodes. Analyzing these genes together with the STRING interactome to construct a PPI network resulted in a primary subnetwork with 1089 nodes, 1644 edges, and 70 seeds (Fig. 1E). The numbers of nodes, edges, and seeds in all other subnetworks are displayed in Supplementary Data 4. The PPI network identified several pathways to be interacting with one another, suggesting that they may function together. Notably, as in the previous Enrichr analysis, the ubiquitin mediated proteolysis pathway was also highlighted here, suggesting its possible involvement in HIV-1 latency. Altogether, the analyses suggest that the genome-wide CRISPR-Cas9 screen successfully identified a network of factors which may be involved in maintenance of HIV-1 latency.

### Functional investigation of putative HIV-1 latency host factors

To further investigate the hits that were obtained in the genome-wide screen, we selected 52 candidate genes based on MAGeCK score, significance in pathway analysis, novelty, and PANTHER Protein Class ontology^36^ for further investigation (Supplementary Fig. 1).

**Figure 2:**
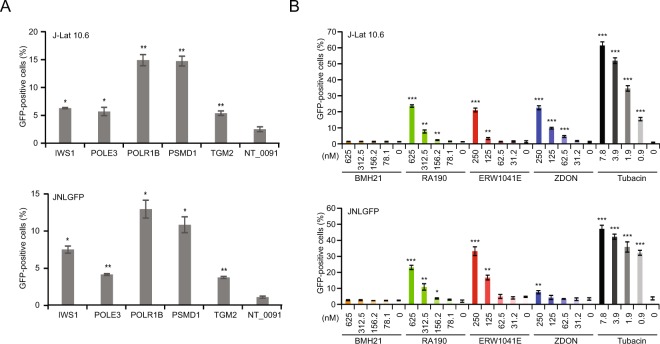
Genetic and pharmacologic investigation of HIV-1 latency-associated candidate genes identified in the CRISPR screen. (**A**) Genetic investigation of selected hits from the primary screen (see Supplementary Fig. 2. for data for all top 50 candidates). Cells were transduced with a single sgRNA cloned into lentiCRISPRv2 targeting the indicated genes in J-Lat 10.6 and JNLGFP cells. GFP-positive cells were quantified by flow cytometry 1 week after puromycin selection. Data are shown as mean ± SD for three experimental replicates. Significance was determined by two-tailed student’s *t*-test comparing each sample against the non-targeting sgRNA control and is denoted as * for *P* ≤ 0.05, ** for *P* ≤ 0.01. (**B**) Pharmacologic investigation of the targets which were verified genetically. J-Lat 10.6 and JNLGFP cells were treated with various inhibitors at the indicated concentrations for 48 h. Tubacin (an HDAC6 inhibitor) served as a positive control. Treated cells were then quantified by flow cytometry to determine the percentage of GFP-positive cells. Data are shown as mean ± SD for three experimental replicates. Significance was determined by two-tailed student’s *t*-test comparing each drug treatment against a DMSO control and is denoted as * for *P* ≤ 0.05, ** for *P* ≤ 0.01, *** for *P* ≤ 0.001.

To confirm these chosen 52 genes, we synthesized and cloned the best sgRNA sequence targeting each gene from the primary screen into a Cas9-expressing vector (lentiCRISPRv2) and transduced them into the J-Lat 10.6 cell line. A non-targeting sgRNA also from the screen was used as a negative control. To rule out cell-line-specific effects, all transductions were also conducted simultaneously in another HIV-1 latency model, the JNLGFP cell line. We found that sgRNAs targeting IWS1, POLE3, POLR1B, PSMD1, and TGM2 resulted in HIV-1 reactivation, as indicated by GFP expression (Fig. 2A, Supplementary Fig. 2). These genes were further validated by utilizing additional sgRNAs which were distinct from the primary screen. These sgRNAs also resulted in similar levels of HIV-1 reactivation in both cell lines as the original sgRNAs (Supplementary Fig. 3, Supplementary Data 5). We found no cell-line-specific differences in reactivation efficiencies of sgRNAs.

In addition to a genetic approach, we sought to further investigate several of the identified host factors through pharmacological modulation. To do so, we tested the small molecule inhibitors BMH21 (POLR1B inhibitor)^37^, RA190 (ADRM1 (PSMD1 interacting partner) inhibitor)^38^, ERW1041E^39^ and ZDON^40^ (TGM2 inhibitors) for reversal of HIV-1 latency in the J-Lat 10.6 and JNLGFP cell lines. The previously identified HIV-1 latency reversal agent (LRA) tubacin (HDAC6 inhibitor) was used as a positive control^41^. RA190, TGI, and ZDON significantly reactivated latent HIV-1 in both of the cell models (Fig. 2B). These inhibitors each reactivated HIV-1 up to 20 to 30-fold at their highest respective concentrations in J-Lat 10.6 with less than 30% toxicity (Supplementary Fig. 4). BMH21 failed to reactivate HIV-1 in either cell line even at the highest concentration. Thus, through genetic and pharmacological perturbations, the previously identified candidates PSMD1 and TGM2 from the primary screen may be involved as HIV-1 latency host factors.

### Deubiquitinase knockout screen identifies additional novel factors in HIV-1 latency

**Figure 3:**
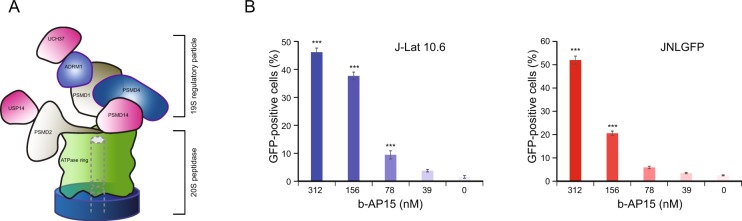
Pharmacologic perturbation of UCH37 deubiquitinase activity reverses HIV-1 latency. (**A**) Model of the typical 26S proteasome, denoting the interacting proteins. PSMD1 interacts with UCH37 through ADRM1. (**B**) Pharmacologic inhibition of UCH37 with b-AP15 in J-Lat 10.6 and JNLGFP cells reverses latency. Cells were treated with b-AP15 at varying concentrations for 48 h before being quantification of HIV-1 latency reversal with flow cytometry. Data are shown as mean ± SD for three experimental replicates. Significance was determined by two-tailed student’s *t*-test comparing each sample against the non-targeting sgRNA control and is denoted as ** for *P* ≤ 0.01, *** for *P* ≤ 0.001.

Of the potential genes, we decided to characterize the role of PSMD1 in HIV-1 latency as genetic knockout and pharmacological inhibition of its interacting protein ADRM1 exhibited the highest magnitude of HIV-1 reactivation. As PSMD1 (scRpn2) encodes for the largest non-ATPase subunit of the 19S regulator lid of the 26S proteasome and this is involved in binding to the deubiquitinase UCH37 (scUCHL5) via the adapter protein ADRM1 (scRpn13), we hypothesized that knockout of PSMD1 may be disrupting the binding of UCH37 to the 26S proteasome, thereby inhibiting its deubiquitinase activity and leading to HIV-1 reactivation^42^ (Fig. 3A).

Although UCH37 was not a hit in the original genome-wide screen, as pharmacological inhibition of ADRM1 by RA190 reversed latency, we wondered whether a similar approach targeting UCH37 would have an effect. Using b-AP15, an inhibitor of UCH37^43^, we found dose-dependent reactivation of HIV-1 in both of the tested cell lines, contrary to the primary screen (Fig. 3B).

**Figure 4:**
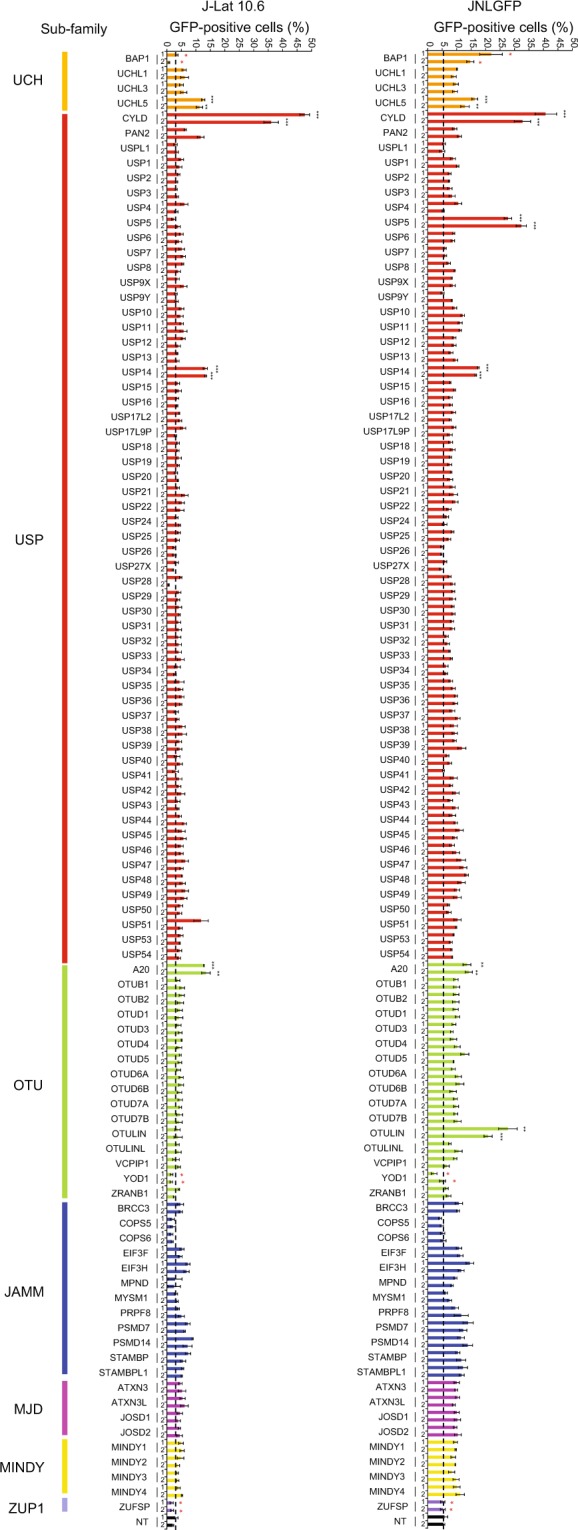
CRISPR-Cas9 knockout screen of deubiquitinases identifies novel negative regulators of HIV-1 latency in cell line models. A sgRNA library targeting 98 deubiquitinase genes (two sgRNAs for each) was designed and cloned into the lentiCRISPRv2 vector for transduction of J-Lat 10.6 and JNLGFP cells. Two non-targeting sgRNAs were used as a control. Cells were then selected for with puromycin for 1 week before quantification with flow cytometry. Deubiquitinases are color-coded by family. Data are shown as mean ± SD for three experimental replicates. Significance was determined by two-tailed student’s *t*-test comparing each sample against the non-targeting sgRNA control and is denoted as ** for *P* ≤ 0.01, *** for *P* ≤ 0.001. SgRNAs denoted by red asterisks exhibited significant cellular toxicity and were excluded from further analysis. Dotted black line indicates background levels of GFP expression. Abbreviations: UCH – Ubiquitin C-terminal Hydrolases, USP – Ubiquitin-specific Proteases, OTU – Ovarian Tumor Proteases, JAMM – JAB1/MPN/Mov34 metalloenzyme (JAMM) motif proteases, MJD – Machado-Joseph disease (MJD) protein domain proteases, MINDY – MIU-containing novel DUB family, ZUP1 – Zinc finger-containing ubiquitin peptidase 1.

Encouraged by those data, we decided to systematically investigate the involvement of deubiquitinases in HIV-1 latency. We conducted a secondary screen by individually knocking out 98 deubiquitinases spanning seven subfamilies. Two sgRNAs targeting each gene were designed (Supplementary Data 6), cloned into lentiCRISPRv2, then transduced into the J-Lat 10.6 and JNLGFP cell lines to examine the reactivation of latent HIV-1 (Fig. 4).

Knockout of several previously known HIV-1 latency factors resulted in HIV-1 reactivation, serving as positive controls for the screen. These included A20 and CYLD, which had robust reactivation in both cell lines as expected^44,45^. In addition to these genes, our screen identified three novel deubiquitinases, OTULIN, USP5, and USP14, which may also be involved in HIV-1 latency. However, we found that knockout of OTULIN and USP5 only resulted in HIV-1 reactivation in the JNLGFP cell line. This cell-line specificity may indicate a mechanism that is dependent on the genetic background of the integrated provirus or that there are different mechanisms of latency in these cell lines.

### Deubiquitinase inhibitors reactivate latent HIV-1

Motivated by the identification of novel deubiquitinases involved in HIV-1 latency via genetic knockout, we investigated whether pharmacological modulation of deubiquitinases could also reactivate latent HIV-1. We tested the latency reactivation efficiency of six deubiquitinase inhibitors, which included specific and broad inhibitors: capzimin^46^ and thiolutin^47^ (PSMD14 inhibitors), WP1130 (USP5/UCHL1/USP9X/USP14/UCH37 inhibitor)^48^, P5091 (USP7 inhibitor)^49^, IU1 (USP14 inhibitor)^50^, and TCID (UCHL3 inhibitor)^51^. As before, both J-Lat 10.6 and JNLGFP cell lines were used for testing of the inhibitors.

**Figure 5:**
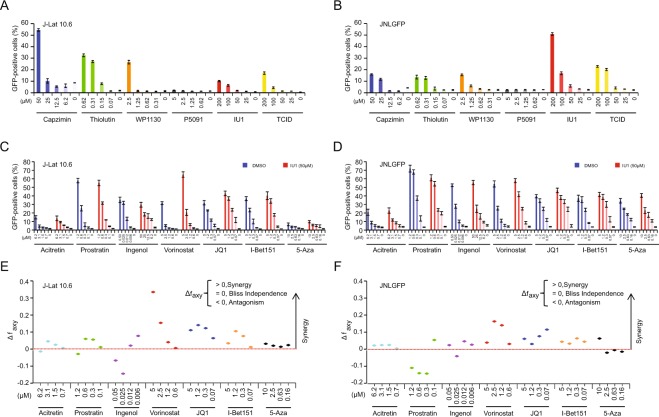
Reactivation of latent HIV-1 in latently infected cells by deubiquitinase inhibitors. Deubiquitinase inhibitors reactivate latent HIV-1. (**A**) J-Lat 10.6 and (**B**) JNLGFP cells were treated with the indicated inhibitors at increasing concentrations for 72 h to examine HIV-1 reactivation capability. GFP-positive cells were quantified by flow cytometry. The indicated drug concentrations did not cause more than 30% toxicity. Data are shown as mean ± SD for three experimental replicates. Significance was determined by two-tailed student’s *t*-test comparing each drug treatment against a DMSO control and is denoted as ** for *P* ≤ 0.01, *** for *P* ≤ 0.001. IU1 synergizes with other LRAs in reactivating latent HIV-1. (**C**) J-Lat 10.6 and (**D**) JNLGFP cells were treated with indicated concentrations of acitretin, prostratin, ingenol, vorinostat, JQ1, I-Bet151, and 5-Aza (5-azacytidine) alone or in combination with IU1 (50 μM) for 48 h before measurement of the percentage of GFP-positive cells by flow cytometry. (**E**,**F**) Synergy of the different LRA combinations was calculated by the Bliss independence model (see Materials and Methods). The dotted horizontal line indicates a purely additive effect (e.g., Δf_axy_ = 0). Synergy is defined as Δf_axy_ > 0 and antagonism is defined as Δf_axy_ < 0. Data are shown as mean ± SD for three experimental replicates.

HIV-1 reactivation was observed in a dose-dependent manner with usage of many of the deubiquitinase inhibitors, including capzimin, thiolutin, WP1130, IU1, and TCID in both J-Lat 10.6 and JNLGFP cell lines (Fig. 5A,B). P5091 did not show reactivation at the concentrations tested. Of note, these drug concentrations caused no more than 30% cell death even under the highest concentrations used (Supplementary Fig. 5).

Given the potential utility of these inhibitors, we next explored whether a synergistic effect could be seen when utilized with currently available latency reversal agents (LRAs). We chose to conduct this analysis with IU1 as it has been previously well-characterized and has high specificity for USP14^50^. The following LRAs which covered a wide range of categories were used: acitretin (RIG-I inducer), prostratin and ingenol (PKC agonists), vorinostat (HDAC inhibitor), JQ1 and I-Bet151 (BET inhibitors), and 5-azacytidine (DNA methyltransferase inhibitor). J-Lat 10.6 and JNLGFP cells were co-treated with IU1 (50 µM) and LRAs at varying concentrations, and then GFP expression was quantified (Fig. 5C,D). Synergistic effects of the drugs were then determined using the Bliss independence model for combined drug effects (Fig. 5E,F)^52^. In both cell lines, we found that IU1 demonstrated modest synergy with vorinostat, JQ1, and I-Bet151, but not with acitretin, prostratin, ingenol, or 5-azacytidine.

### IU1 reactivates latent HIV-1 without global T cell activation

To determine the clinical relevance of IU1, we asked whether IU1 was coupled to global T cell activation. Purified primary CD4^+^ T cells were treated with 200 µM of IU1, 1 µM of b-AP15, or 0.01 µM of romidepsin for 24 h and the expression of global T cell activation markers CD25 and CD69 was measured by flow cytometry. Expression of CD25 was not altered after treatment with any of the inhibitors. As previously observed^53^, romidepsin treatment showed enhanced expression of CD69 compared to DMSO-treated control cells, whereas IU1 and b-AP15 did not alter the surface expression of CD69 (Supplementary Fig. 6). The lack of global T cell activation suggests the potential of deubiquitinase inhibitors such as IU1 as a HIV-1 LRA.

### TDP-43 depletion reactivates latent HIV-1

**Figure 6:**
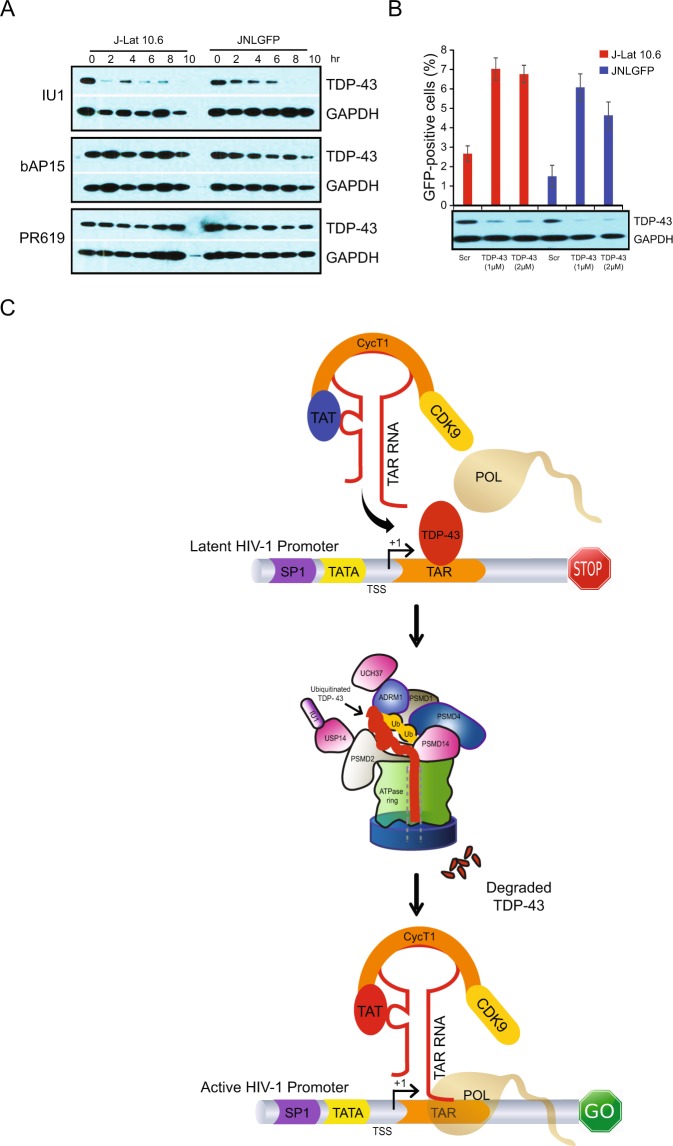
TDP-43 knockdown reverses HIV-1 latency. (**A**) IU1 causes degradation of TDP-43. J-Lat 10.6 and JNLGFP cells were treated with IU1 (200 μM) and expression of TDP-43 at the indicated time points was determined by immunoblot. GAPDH was used as a loading control. (**B**) Knockdown of TDP-43 reactivates HIV-1. J-Lat 10.6 and JNLGFP cells were treated with a control siRNA (Ctrl) or with a pool of four different TDP-43-targeting siRNAs at 1 μM or 2 μM. Cells were then quantified by flow cytometry for GFP expression or by immunoblot for TDP-43 expression. Data are shown as mean ± SD for three experimental replicates. GAPDH was used as a loading control for the immunoblot. Abbreviations: Scr – scrambled non-targeting control siRNA pool. (**C**) A model for the IU1-mediated reactivation of HIV-1. TDP-43 (blue) can bind to the LTR promoter of HIV-1 to prevent reactivation by steric hindrance. Inhibition of USP14 deubiquitinase activity by IU1 causes enhanced degradation of its substrate proteins, such as TDP-43. This degradation then frees the LTR for binding by factors such the SEC complex, thereby inducing transcription of latent HIV-1.

We next interrogated the mechanism of IU1-mediated HIV-1 reactivation. As previous studies showed that IU1 promotes degradation of several endogenous proteins, including TDP-43^50^, a transcriptional repressor which binds to the HIV-1 trans-active response element (TAR)^54,55^. Therefore, we hypothesized that IU1-mediated TDP-43 degradation might cause HIV-1 reactivation. To test our hypothesis, we treated J-Lat 10.6 and JNLGFP cells with IU1 (200 µM) and analyzed TDP-43 protein expression at different time points. We observed degradation of endogenous TDP-43 within 2–6 h of IU1 treatment in both cell line models (Fig. 6A, Supplementary Fig. 7). In contrast, b-AP15 and PR619 treatment showed no effect on TDP-43 in either cell line.

As further confirmation, we directly tested the role of TDP-43 in HIV-1 latency by knocking down expression of TDP-43 with siRNAs. Knockdown of TDP-43 in both J-Lat 10.6 and JNLGFP cell lines with two different concentrations of siRNA resulted in a significant increase in reactivation compared to a scrambled negative control siRNA (Fig. 6B). Taken together, these results indicate IU1 may function by relieving the steric hindrance posed by TDP-43 binding to the HIV-1 LTR promoter, thereby inducing transcription and viral reactivation (Fig. 6C).

## Discussion

Host factors involved in HIV-1 latency have been extensively studied over the past decades^56^. Yet, a full understanding of the underlying mechanisms involved in HIV-1 latency remains elusive, and consequently attempts at therapeutic reactivation of latent HIV-1 have not been successful^15^. Here, to identify novel latency promoting genes, we undertook a whole genome CRISPR-Cas9 pooled knockout screen in J-Lat 10.6 cells, a Jurkat-based T-cell model of HIV-1 latency. This comprehensive approach revealed several genes to be involved in latency, including several that were novel (POLE3, POLR1B, and TGM2), well-characterized (IWS1)^57^, or recently reported (PSMD1)^22^.

Two candidates from our screen were POLE3 and IWS1, reported to be a part of heterochromatin remodeling complex^57,58^. POLE3 (DPB4/YBL1/CHRAC17) is a subunit of the DNA polymerase ε, which selectively binds to histones H3-H4 during replication-coupled nucleosome assembly^58^. In this role, POLE3 is important for chromatin maintenance and new histone deposition, and it can be speculated that the genome, including integrated HIV-1 provirus, would be more accessible for transcription in the absence of histone deposition. IWS1 is also a member of the remodeling complex and has been previously shown to negatively regulate HIV-1 latency through binding to the LEDGF/p75/Spt6 complex^57^. It has been suggested that IWS1 affects the histone modification state of actively transcribed HIV-1, thereby acting as a transcriptional repressor^57^. Therefore, small molecules that can target these complexes could provide a novel strategy for HIV-1 reactivation.

POLR1B is involved in ribosome biogenesis as a core component of RNA polymerase I^59^. Silencing of POLR1B has been previously shown to lead to the downstream activation of p53 through the RP-MDM2-p53 stress response pathway^60^. Activation of such a pathway would be expected to lead to global gene reactivation including that of HIV-1. Intriguingly, pharmacological inhibition of POLR1B through BMH-21, which blocks Pol I transcription, did not show reactivation of HIV-1, despite the effects observed with the genetic knockout. This difference observed here between the genetic and pharmacological inhibition of POLR1B suggests that further studies will be required to fully elucidate the mechanism by which it may be involved in HIV-1 latency.

Another interesting candidate we discovered in our screen was TGM2, a multifunctional enzyme which cross-links proteins by catalyzing an acyl transfer reaction between glutamine and lysine residues^61^. We found that genetic knockout and pharmacologic inhibition by TGM2 inhibitors (ZDON and ERW1041E) reversed HIV-1 latency. Previous reports have suggested a role for TGM2 in the suppression of HIV-1 replication by altering the viral mRNA trafficking between the nucleus and the cytoplasm^62^. In other contexts, TGM2 has been shown to be involved in transcriptional repression, with inhibition of this enzyme resulting in specific increase in mRNA levels^63^. It is also noteworthy that TGM2 has been shown to be involved in the resistance of cancer to vorinostat, an HDACi that has also been tested for HIV-1 latency reversal^64^. Collectively, these studies and our data indicating HIV-1 latency reversal by genetic or pharmacological inhibition of TGM2, suggests that it could be a novel target for reactivation of HIV-1.

PSMD1 is the largest 26S proteasome non‐ATPase 19S regulatory subunit, mediating the degradation of proteins that have been targeted for destruction by ubiquitination^65^. Recently, PSMD1 was identified in a genome-wide CRISPRi screen as a novel host factor that inhibits Tat-dependent HIV-1 transcription^22^. This study demonstrated that PSMD1 knockdown significantly increased HIV-1 LTR-dependent transcription; however, its precise role in HIV-1 latency was not fully characterized. Although it is suggested that downregulating PSMD1 causes inhibition of the proteasome, thereby enabling Tat-transactivation, the effect of PSMD1 depletion on proteasomal activity was not verified. We speculated that it is also plausible that as PSMD1 serves as a binding site for other subunits to the proteasome, the reversal of HIV-1 latency may be through disruption of these interactions. For example, PSMD1 interacts with ADRM1, which recruits and activates UCH37, a deubiquitinating enzyme^42^. We tested this possibility through pharmacological inhibition of the ADRM1-PSMD1-UCH37 interaction by using RA190^38^. We found that in two different HIV-1 latency cell lines, RA190 induced reactivation. As RA190 has also been proposed to specifically inhibit UCH37 in addition to disrupting the above interaction, we speculated that targeting UCH37 directly could also reverse HIV-1 latency. We investigated this hypothesis with use of the inhibitor b-AP15, which also caused reactivation, although this inhibitor acts on both UCH37 and another deubiquitinase, USP14^66^.

The success of inhibiting the above deubiquitinases for latency reversal suggested that there may be a direct role for these enzymes in HIV-1 latency. In particular, given the critical role of deubiquitinases in negatively regulating NFκB signaling, it is expected that they could be involved in regulating HIV-1 latency^67^. Indeed, several studies have examined this relationship, identifying that deubiquitinases such as CYLD^27,45^ and ABIN1^44^ promote HIV-1 latency. We systematically knocked out 98 deubiquitinases using CRISPR-Cas9 in two different cell lines to investigate their role in HIV-1 latency. We observed some differences in the results between the two models, likely due to the intrinsic differences between the viruses used to derive each system. J-Lat 10.6 was made from an HIV-1 expressing GFP, with a frameshift mutation in *env* and deletion of the *nef* gene^29^. In contrast, the JNLGFP cell line was derived from a replication competent HIV-1 GFP reporter virus^68^. Regardless, as expected, our screen identified deubiquitinases previously reported to be involved in the negative regulation of NFκB (CYLD, A20, and OTULIN)^67^. Furthermore, we discovered new deubiquitinases to be involved in promoting HIV-1 latency: UCH37, USP5, and USP14.

These newly identified deubiquitinases were further investigated through pharmacological inhibition. As previously mentioned, the dual inhibitor of UCH37 and USP14, b-AP15, reversed HIV-1 latency. In addition, the specific inhibitor of USP14, IU1, reversed HIV-1 latency, suggesting that targeting USP14 alone can reactivate latent HIV-1^50^. Interestingly, pharmacological inhibition of PSMD14 with capzimin and thiolutin and UCHL3 with TCID reversed HIV-1 latency, although genetic knockout of these deubiquitinases did not^46,47^. This suggests that these compounds may have unreported off-target activity, or that the genetic knockout was incomplete.

Given that targeting of USP14 by both genetic and pharmacological methods reversed HIV-1 latency, we conducted further investigation of its specific inhibitor, IU1. This inhibitor demonstrated synergistic HIV-1 reactivation when used with other LRAs, including vorinostat, JQ1, and I-BET151, indicating that it may utilize a pathway differing from these compounds. IU1 has previously been shown to target the USP14 catalytic site and promote degradation of several overexpressed proteins, including TDP-43^50^. As TDP-43 has been identified as a repressor of HIV-1 transcription, we hypothesized that it may be targeting of this protein by IU1 that reverses latency^54^. Indeed, we found time-dependent degradation of endogenous TDP-43 in both J-Lat 10.6 and JNLGFP cells after IU1 treatment. Moreover, knockdown of TDP-43 by shRNAs in both cell lines also reactivated latent HIV-1. Future experiments remain to be conducted to investigate how TDP-43 depletion or overexpression may affect IU1-induced activation of HIV-1 transcription to thoroughly investigate the mechanism by which IU1 operates on latent HIV-1. It may also be worth pursuing how HIV-1 proviruses that have a mutated TAR region at TDP-43-binding sites respond to treatment by IU1. We note that this contrasts a previous report which investigated TDP-43 as a restriction factor and found that TDP-43 did not affect HIV-1 gene expression in T cells and macrophages^69^. However, this study evaluated TDP-43 as a restriction factor, and not as a latency promoting factor, an important distinction. Such differences have been previously shown with other proteins; for example, while APOBEC3G is a well-characterized restriction factor for HIV-1, its knockdown or knockout does not reverse HIV-1 latency^70^. Altogether, while additional experiments in primary human CD4^+^ T cell models of HIV latency or in cells from cART-suppressed HIV-1 infected patients are required to fully elucidate the mechanism of IU1-mediated HIV-1 reactivation, targeting of USP14 holds potential for HIV-1 latency reversal.

In summary, this study represents the first comprehensive evaluation of the deubiquitinase family as HIV-1 latency promoting factors. Several novel deubiquitinases were identified and targeting of these enzymes through pharmacological inhibition was shown to reverse HIV-1 latency. Collectively, these findings suggest that altering cellular proteostasis through the targeting of deubiquitinases may be a promising avenue for the development of latent HIV-1 interventions. As it becomes increasingly likely that a multitude of approaches and targets will be necessary for full reversal of HIV-1 latency, investigation of such additional pathways remains crucial.

## Materials and Methods

### Cell culture

The HIV-1 latency cell line J-Lat 10.6 was obtained from the NIH AIDS Reagent Program (Catalog #9849)^29^ and the JNLGFP cell line was a kind gift from Dr. David N. Levy (New York University, New York, NY)^68^ and cultured in RPMI-1640 medium with 10% fetal calf serum (FCS). 293FT cells (Invitrogen) were cultured in DMEM (Life Technologies) and supplemented with 10% heat-inactivated FCS (Sigma), 2 mM glutamine, and antibiotics (50 U/mL penicillin and 50 mg/mL streptomycin). All cultures were maintained at 37 °C, 5% CO_2_, and 95% humidity.

### J-Lat 10.6_Cas9 cell line generation

J-Lat 10.6 cells were stably transduced with lentiCas9-Blast (Addgene #52962)^30^. Following 1 week of blasticidin (Lifetech) selection at 10 µg/mL, single, viable, GFP-negative cells were sorted into 96-well U bottom plates by a Beckman Coulter MoFlo XDP flow sorter. Sub-clones were analyzed by western blot for expression of Cas9 (Diagenode, C15200203). For knockout screening, a sub-clone was selected for high Cas9 expression and functional activity, as well as low basal GFP expression. Functional activity of Cas9 was determined by loss of GFP-positive cells in the presence of PXPR_011 (Addgene #59702) plasmid, described in detail elsewhere^71^.

### CRISPR Knock-Out library amplification and lentiviral production

The human GeCKO v2 “A” and “B” library pooled plasmid (lentiCRISPRv2) was gift from Feng Zhang (Addgene #1000000048) and amplified according to the recommended protocol^72^. Briefly, plasmids were electroporated into Lucigen Endura electrocompetent cells, expanded to a 150 mL culture, and isolated by maxiprep (Zymo Research #D4203). Lentiviral particles were produced by co-transfecting 293FT cells with 12 μg of GeCKO plasmid library, 9 μg of packaging plasmid psPAX2 (Addgene #12260) and 6 μg of VSV-G-expressing envelope plasmid pMD2.G (Addgene #12259) in 15-cm plates using 54 μL of Lipofectamine in Opti-MEM medium. The supernatant was harvested after 48 h, centrifuged at 3,000 RPM at 4 °C for 10 min, filtered through a 0.45 μm low protein-binding membrane (Millipore), and concentrated by ultracentrifuging through a sucrose gradient method at 22,000 RPM at 4 °C for 2 h. The virus was then aliquoted and frozen at −80 °C.

### Pooled genome-wide CRISPR screen

We followed previously published protocols of screens using the GeCKO library, as developed by the Zhang Lab, with slight modifications^72^. Approximately 30 million J-Lat 10.6_Cas9 cells that constitutively express Cas9 were transduced with lentiviruses derived from the lentiGuide-Puro construct from the GeCKO v2_A/B at an MOI of 0.3. After 21 days of puromycin selection, the transduced cells were split into two groups of approximately 10 million cells. One group was used for sorting GFP-positive cells and other group was left unsorted. Two replicates for each library was used in the study. For both GFP sorted experimental and unsorted control cells, gDNA was isolated using a QIAamp DNA column and the inserted guide RNA sequences were amplified from the gDNA by flanking primers and prepared for next-generation sequencing. All libraries were pooled and sequenced on HiSeq instruments using 1000-bp single-end reads (Illumina) at The Rockefeller University Genomics Resource Center. The sgRNA reads were further de-convoluted and enrichment of each guide RNA was calculated by comparing the relative abundance in the selected and unselected population. Relative enrichment or depletion of sgRNAs and genes was analyzed using combined analysis of both GeCKO v2_A and B sgRNA using the MAGeCK statistical package^31^.

### Single gene validation knock-out

Following the latency screen, top candidate genes were tested for validation by using two independent sgRNAs per gene from the parent library and cloning them into the plasmid lentiCRISPRv2 (Addgene #52961)^30^. The control sgRNAs were used from the parent library. Similarly for the DUB knockout screen, sgRNAs targeting deubiquitinases were designed and cloned into the lentiCRISPRv2 vector and confirmed by sequencing according to the Zhang Lab protocol^30^.

### HIV-1 latency reversal by drug treatments *in vitro*

50,000 J-Lat 10.6 and JNLGFP cells were aliquoted into 96-well plates in 200 μL medium. Each of the following drugs were added to the cells in triplicate at the indicated concentrations: BMH-21 (MedKoo, 406586), RA190 (Calbiochem, 5.30341.0001), ERW1041E (Sigma, 509522), ZDON (Calbiochem, 616467), ZDON (Calbiochem, 15227), tubacin (Sigma, SML0065), b-AP15 (Cayman chemical, 11324; AdooQ Bioscience, A15391; MedKoo, 406452), capzimin (Glix labs, GLXC-09966), thiolutin (MedKoo, 525293; Cayman chemical, 11350), WP1130 (Cayman chemical, 15227), P005091 (MedKoo, 406577; Cayman chemical, 15224), IU1 (AdooQ, A13209; Cayman chemical, 10617), TCID (Cayman chemical, 16353; Sigma, SML1402), acitretin (Sigma, 44707), prostratin (Sigma, P0077), ingenol (Sigma, SML1318), vorinostat (Sigma, SML0061), JQ1 (Sigma, SML1524), I-BET151 (Tocris, 4650), 5-azacytidine (Sigma, A3656), romidepsin (Sigma, SML1175), PR619 (AdooQ Bioscience, A13190). For control groups, 0.1% DMSO was used. After 48 or 72 h of treatment, GFP expression in the cells were measured by flow cytometry. Cell viabilities were determined by forward scatter vs. side scatter gating using untreated cells as the control. The data were analyzed with FlowJo software and plotted as bar graphs.

### Flow cytometry

For high throughput quantification of GFP positive cells, a Guava® EasyCyte flow cytometer was used. For each individual sgRNA knockout validation, 10,000 cells were used. For cell surface marker analysis, data was acquired on a BD LSR II (Becton Dickinson) and analyzed using FlowJo software (FACSDiva). Cell viability was monitored by forward-and-side scatter analysis and data was analyzed using FlowJo 9.9 (Tree Star). Cell sorting was performed by Flow Sorter Beckman Moflo XDP (Beckman Coulter).

### PBMC isolation and cell activation analysis

PBMCs from healthy donors were isolated by Ficoll gradient centrifugation from whole blood purchased from the New York Blood Center. Isolated cells were cultured in complete RPMI media, consisting of RPMI-1640 supplemented with 5 mM HEPES, 2 mM Glutamine, 50 μg/mL penicillin/streptomycin, 5 mM nonessential amino acids, 5 mM sodium pyruvate, and 10% fetal bovine serum (Thermo Fisher). PBMCs were incubated for 24 h in 6-well plates with IU1 (200 μM), b-AP15 (1 μM), or Romidepsin (0.1 μM). After washing with FACS buffer (PBS, 2% FBS), PBMCs were stained with the following cocktail of anti-human antibodies: CD3-FITC (BioLegend, San Diego, CA), CD4-Pacific Blue (BioLegend), CD25-PE-Cy7 (BioLegend), and CD69-Alexa Fluor 700 (BioLegend). In addition, LIVE/DEAD Yellow stain (Invitrogen, Thermo Fisher Scientific, Waltham, MA) was used to exclude dead cells. Staining was performed for 30 min on ice in FACS buffer. After washing, the samples were analyzed on a multi-laser flow cytometer LSR-II (BD Bioscience, San Jose, CA). Fluorescence compensation was performed with anti-mouse IgG (k) beads (BD Biosciences) stained with each antibody in a separate tube. The total content of PBMCs acquired by BD LSRII was analyzed with FlowJo (TreeStar, Cupertino, CA).

### TDBP43 gene silencing by RNA interference

500,000 HIV-1 latent cells in 1 mL of Accell siRNA delivery medium (Thermo Scientific Dharmacon) were cultured in a 12-well tissue culture plate (Nunc). Commercially available Accell TARDBP siRNA smart pool (Thermo Scientific Dharmacon, E-012394) was reconstituted in 1× siRNA buffer (Thermo Scientific Dharmacon) to a stock concentration of 100 μM. From this stock solution, 10 μL or 20 μL of TARDBP siRNA was added to each well and mixed gently. The cells were incubated at 37 °C under 5% CO_2_ for 72 h. Accell non-targeting siRNA #1 (Thermo Scientific Dharmacon, D-001910) control was used as a negative control. After 72 h, a portion of the cells were quantified for GFP expression by BD LSRII and analyzed with FlowJo (TreeStar, Cupertino, CA) while the remainder was assayed for TDP-43 (anti-TARDP43, sc376532) and GAPDH (anti-GAPDH, sc-47724) expression by immunoblot as described below.

### Western blotting

Cells were rinsed once with ice-cold PBS and immediately lysed with 2X Laemmli sample buffer (BioRad, 161-0737) containing 355 mM of 2-mercaptoethanol (Bio-Rad, 1610710). The cell lysates were boiled at 100 °C for 10 min, vortexed and spun down, loaded onto and separated on a NuPAGE Novex 12% Tris-Glycine gel, then transferred to a polyvinylidene difluoride membrane (Millipore). Immunoblots were processed according to standard procedures, using primary antibodies directed to TDP-43 and GAPDH and analyzed using enhanced chemiluminescence (Clarity^TM^ Western ECL Substrate, Bio-Rad, 1705060), with HRP-conjugated anti-mouse and anti-rabbit secondary antibodies (Santa Cruz Biotechnology).

### Statistical analysis

Differences between groups were analyzed for statistical significance using the two-sample unequal variance students T-test distribution. *P* values < 0.05 were considered statistically significantly different. The Bliss independence model was utilized to determine synergism/antagonism of drug combinations^52^.

## Supplementary information

SUPPLEMENTARY INFORMATION.

Dataset 1.

Dataset 2.

Dataset 3.

Dataset 4.

Dataset 5.

Dataset 6.

## Data Availability

All relevant data are available from the authors on request.
