# Supplementary material for: CRISPR-based gene knockout screens reveal deubiquitinases involved in HIV-1 latency in two Jurkat cell models
Source: Sci Rep. 2020 Mar 24;10:5350. doi: 10.1038/s41598-020-62375-3 (PMC7093534; doi:10.1038/s41598-020-62375-3)

**CRISPR-based gene knockout screens reveal deubiquitinases involved in HIV-1 latency in two Jurkat cell models**

Anurag Rathore^1^, Sho Iketani^1, 2^, Pengfei Wang^1^, Manxue Jia^1^, Vincent Sahi^1^,
and David D. Ho^1*^

^1^ Aaron Diamond AIDS Research Center, Columbia University Irving Medical Center, New York, NY 10032, USA ^2^ Department of Microbiology and Immunology, Columbia University Irving Medical Center, New York, NY 10032, USA

Running title: Role of deubiquitinases in HIV-1 latency

*Correspondence: dh2994@cumc.columbia.edu (D.D.H.)

**Supplementary Information**

**Supplementary Fig. 1.** Top 50 candidate genes selected by enrichment in a relevant biological pathway, protein-protein network analysis, literature search, and panther protein class category.

**Supplementary Fig. 2.** J-Lat 10.6 and JNLGFP cells were transduced with Cas9 and with the sgRNA with the highest enrichment in the previous analysis. Cells were selected for with puromycin for 1 week, and then GFP-positive cells were quantified by flow cytometry. SgRNAs showing greater than 30% toxicity were excluded from the analysis. Candidates genes are grouped according to panther protein class category and data are shown as mean ± SD for three experimental replicates. Dotted red line indicates background levels of GFP expression.

**Supplementary Fig. 3.** Validation of IWS1, POLE3, POLR1B, PSMD1, and TGM2 as HIV-1 latency factors with additional sgRNAs. SCR denotes scrambled non-targeting sgRNA.

**Supplementary Fig. 4.** Confirmation of the identified hits POLR1B, PSMD1, and TGM2 via pharmacological inhibition. Percentage of living cells at indicated drug concentrations were determined by FSC and SSC gating following flow cytometry.

**Supplementary Fig. 5.** Testing of deubiquitinase inhibitors for HIV-1 reactivation. Percentage of living cells at indicated drug concentrations were determined by FSC and SSC gating following flow cytometry.

**Supplementary Fig. 6.** IU1 does not cause global T cell activation. Effect of IU1 on CD25 and CD69 expression. Human CD4^+^ T cells were treated with either IU1 (200 μM), b-AP15 (1 μM), or Romidepsin (0.01 μM) for 24 h and then the expression of CD25 and CD69 was quantified by flow cytometry. (A) Representative FACS plots are shown. Aggregated data of (B) CD25 expression and (C) CD69 expression from two different donors are shown. Data are shown as mean ± SD.

**Supplementary Fig. 7.** Full length western blot image showing (A) TDP-43 and (B) GAPDH as a loading control.

**Supplementary Data 1.** Analysis of the sgRNAs in the primary screen by MAGeCK.

**Supplementary Data 2.** Rank, FDR, log fold change, and P-value of each gene from the primary screen as determined by MAGeCK.

**Supplementary Data 3.** Enrichment analysis of the top 211 candidate genes.

**Supplementary Data 4.** Network analysis to identify protein-protein interactions as defined by the STRING interactome. Degree and betweenness values for the top 211 candidate genes are shown. Degrees are the number of connections that a node has to other nodes and the betweenness values are a measure of the number of shortest paths to the node. Different subnetworks were identified for the genes, with subnetwork 1 being used for **Fig. 1E**.

**Supplementary Data 5.** Sequences of sgRNAs used for validation of the hits from the primary screen.

**Supplementary Data 6.** Sequences of sgRNAs used for the deubiquitinase knockout screen.

**Supplementary Figure 1**

**Supplementary Figure 2**

**Supplementary Figure 3**

**Supplementary Figure 4**

**Supplementary Figure 5**

**Supplementary Figure 6**

**Supplementary Figure 7**


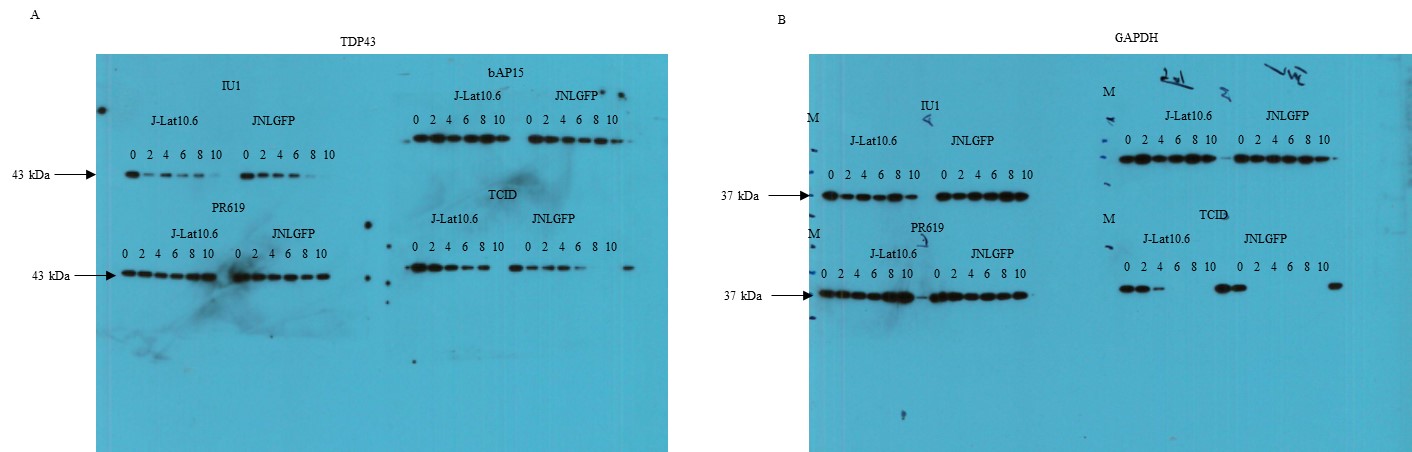

Supplement: Supplementary file 1 — SUPPLEMENTARY INFORMATION. [file 41598_2020_62375_MOESM1_ESM.docx]
